# The Ground-Dwelling Arthropod Community of Península Valdés in Patagonia, Argentina

**DOI:** 10.1673/031.010.5001

**Published:** 2010-05-17

**Authors:** Germán H. Cheli, J. C. Corley, O. Bruzzone, M. del Brío, F. Martínez, N. Martínez Román, I. Ríos

**Affiliations:** ^1^ Unidad de Investigación Ecología Terrestre, CENPAT-CONICET, Bvd. Brown 2915 (9120), Puerto Madryn, Chubut, Argentina; ^2^ Laboratorio de Ecología de Insectos, INTA EEA Bariloche, CC 277 (8400), Bariloche, Río Negro, Argentina; ^3^ Universidad Nacional de la Patagonia San Juan Bosco, Bvd. Brown 3700 (9120), Puerto Madryn, Chubut, Argentina

**Keywords:** abundance, desert, diversity, epigeal arthropods, guild, pitfall trapping

## Abstract

This is the first study based on a planned and intensive sampling effort that describes the community composition and structure of the ground-dwelling arthropod assemblage of Península Valdés (Patagonia). It was carried out using pitfall traps, opened for two weeks during the summers of 2005, 2006 and 2007. A total of 28, 111 individuals were caught. Ants (Hymenoptera: Formicidae) dominated this community, followed by beetles (Coleoptera) and spiders (Araneae). The most abundant species were *Pheidole bergi* Mayr (Hymenoptera: Formicidae) and *Blapstinus punctulatus* Solier (Coleoptera: Tenebrionidae). Two new species were very recently described as new based on specimens collected during this study: *Valdesiana curiosa* Carpintero, Dellapé & Cheli (Hemiptera, Miridae) and *Anomaloptera patagonica* Dellapé & Cheli (Hemiptera, Oxycarenidae). The order Coleoptera was the most diverse taxa. The distribution of abundance data was best described by the logarithmic series model both at the family and species levels, suggesting that ecological relationships in this community could be controlled by a few factors. The community was dominated by predators from a trophic perspective. This suggests that predation acts as an important factor driving the distribution and abundances of surface-dwelling arthropods in this habitat and as such serves as a key element in understanding desert, above-ground community structure. These findings may also be useful for management and conservation purposes in arid Patagonia.

## Introduction

The achievement of a complete inventory of the earth's biota remains an urgent priority for biodiversity conservation. One of the main challenges is exploring the wilder regions of the world where intact habitats of high conservation value remain unknown. Arid areas are a major terrestrial habitat among these environments (Polis 1991).

In South America, deserts are the largest macro-habitat, covering more than 57.3% of the surface area (Mares 1992). The dry neotropics support considerable biological diversity, though they have received little attention in comparison with the wet, tropical forests (Bestelmeyer and Wiens 1996). Patagonia is a large xeric biome located in the southern tip of South America, remarkably understudied despite the fact that some of the original components and functions of this arid ecosystem are still preserved. One of the largest conservation units of arid ecosystems in Argentina is the Natural Protected Area Península Valdés, located in the northeastern zone of this biome. Since 1999, this area has been included in the UNESCO World Heritage List.

Invertebrates represent an essential part of ecosystems (Seymour and Dean 1999) having great abundances and species richness in almost all habitats (James et al. 1999; Andersen et al. 2004; Corley et al. 2006), occurring at all levels of the food web (Samways 1994; Seymour and Dean 1999; Andersen et al. 2004), and playing vital roles in the structure and fertility of soils, the pollination of flowering plants, nutrient cycling, and in the decomposition of organic material and predation (Greenslade 1992; Ayal et al. 2007). Furthermore, arthropods can be used for monitoring environmental changes because of their high species abundances, richness, and habitat fidelity (Andersen and Majer 2004). Terrestrial arthropods are even better monitors than vegetation because of their rapid response to habitat changes and the capability of generating a finer environmental classification than vascular plants or vertebrates (Samways 1994; Seymour and Dean 1999; Andersen et al. 2004).

In arid regions, invertebrates are the most abundant animals (Crawford 1986; Ayal et al. 2007). In these habitats, arthropods play key roles (principally in and above the soil) as decomposers, herbivores, granivores, and predators, controlling nutrient and energy flow through trophic levels in the food chain (Crawford 1986; Polis 1991; Greenslade 1992; Ayal et al. 2007). Arthropods fill these important functional roles in deserts because they are less constrained by low water availability and extreme thermal environments than other animals (Whitford 2000; Andersen et al. 2004). The arthropod biomass and species diversity is much greater than all other desert animal biomass and diversity combined (Polis 1991).

The aim of this work was to give a preliminary description of the composition and structure of the arthropod community of Península Valdés, using species abundance models, diversity analysis and a trophic guild approach, based on a planned and intensive sampling effort. The purpose is to contribute to a currently limited knowledge of the ground-dwelling arthropod fauna of Patagonia (Cuezzo 1998; Flores 1998; Ceballos and Rosso de Ferradás 2008; Crespo and del Valverde 2008; Ocampo and Ruiz Manzanos 2008).

## Materials and Methods

**Figure 1.**
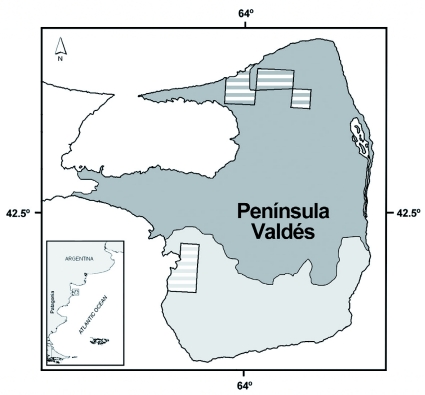
Main environmental units and geographical location of the sampling sites in the study area (dark grey: shrub steppe; light gray: shrub-grass steppe). High quality figures are available online.

Ground-dwelling arthropods were sampled using pitfall traps during the summers of 2005, 2006 and 2007. A total of 648 traps, 12 cm in diameter at the opening and 12 cm deep, were placed (216 traps/year). According to previous optimization studies of the pitfall sampling in the area (Cheli, unpublished observations), each trap was filled with 300 ml of a 30% solution of ethylene glycol used as a preservative, and each trap was opened on-site for two weeks in the middle of February. Traps were located at least 20 m apart from each other, covering the main environmental units of Península Valdés (Figure 1). The two main vegetation units of Península Valdés are: (1) shrub steppe with 67%) of total vegetal cover dominated by *Chuquiraga avellanedae* Lorentz (Asterales: Asteraceae), *Condalia microphylla* Cav. (Rosales: Rhamnaceae), *Paronychia chilensis* DC (Caryophyllales: Caryophyllaceae), *Hoffmanseggia trifoliata* Cav. (Fabales: Fabaceae), *Nassella tenuis* (Phil.) Barkworth (Poales: Poaceae), *Achnatherum speciosa* (Trin, & Rupr.) Barkworth (Poaceae), *Poa ligularis*Nees & Steud. (Poaceae); and (2) shrub-grass steppe with 75%> of total vegetal cover dominated by *C. avellanedae*, *Hyalis argentea* D. Don ex Hook & Arn (Asteraceae), *H. trifoliata*, *P. chilensis*, *S. tenuis*, *Sporobolus rigens* (Trin.) E. Desv. (Poaceae), *Piptochaetium napostaense* (Speg.) Hack. (Poaceae), *Plantago patagonica* Jacq. (Lamiales: Plantaginaceae) (Bertiller et al. 1981).

All specimens were identified to order and family levels. Additionally, in order to have a good estimation of the community structure at the species level, three representative groups with different abundances were chosen: Formicidae (Hymenoptera) (the most abundant taxa), Coleoptera (a medium to high abundance taxon), and Heteroptera (Hemiptera) (low abundance taxa).

In those cases where it was not possible to determine individuals at the species level, the individuals were described as morphospecies for further analysis. Voucher specimens were deposited in the entomological collection of Centro Nacional Patagónico (CENPAT-CONICET), Museo de La Plata and IADIZA (CRICYT-CONICET). Araneae were only analyzed to the order level due to the large numbers of juvenile specimens and of individuals whose small size impeded proper determination. The same level of analysis was used for Psocoptera because of the lack of accurate literature and keys. Finally, flying Hymenoptera, Lepidoptera, and the suborder Auchenorrhyncha (Hemiptera) were excluded from analysis because the sampling protocol used for this study was not suited for these groups.

### Statistical analysis

Abundance analysis: Abundance distribution models were used to describe the structure of the community. To choose which model best described the community, a Bayesian selection was performed for four models. Those models increased in their evenness as follows: (a) Dominance pre-emption model, (b) Logarithmic Series, (c) Logarithmic Normal Distribution, and (d) MacArthur's Broken Stick model (Tokeshi 1990, 1993; Magurran 2004).

The decision criterion for choosing a model was the lowest value of the Akaike Information Criterion (AIC) (Gelman et al. 2003). The estimation of parameters was calculated by means of Markov Chain Montecarlo (Gelman et al. 2003) using the pymc library for Bayesian estimation for the python programming language (Fonnesbeck 2009).

Diversity analysis: Diversity was estimated through the Shannon-Wiener index, the Shannon evenness measure, and the richness of families and species (Moreno 2001; Magurran 2004). The Shannon-Wiener diversity index was calculated using natural log, and differences between groups were tested by the Hutchenson method (a modification of the t-test, see Magurran 1988) using Bio∼DAP software.

Guild analysis: To indicate the trophic structure of the arthropod community, species were classified into feeding guilds as herbivores, predators, and scavengers (following Borror et al. 1989; Morrone and Coscarón 1998; Claps et al. 2008). The relationship among abundance and richness of feeding guilds was analyzed using the *X^2^* test. All α-values for multiple tests were corrected by Bonferroni's correction (α' = α /3 = 0.0167) (Zar 1999).

## Results

A total of 28, 111 arthropods belonging to 18 orders, 52 families and 160 species/morphospecies were collected. At the order level, Hymenoptera (Formicidae and Mutillidae) represented 83.2% of the total catch, thus there were very low relative abundances of other orders.

**Table 1.**
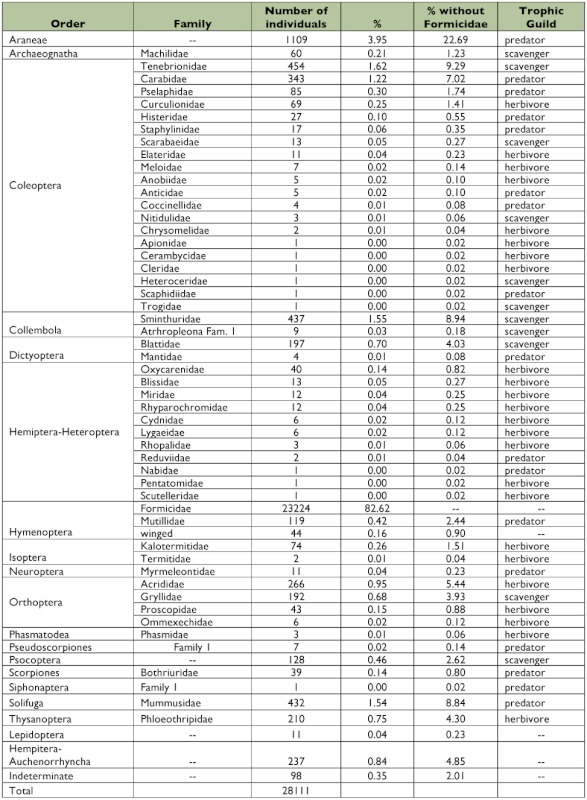
Arthropod orders and families collected through pitfall trapping in Península Valdés.

**Figure 2.**
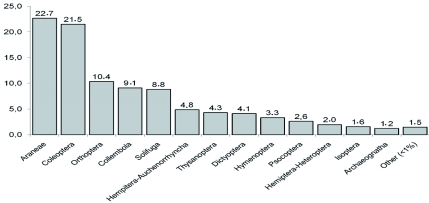
Relative abundance (%) of orders collected from Península Valdés (Patagonia, Argentina).High quality figures are available online.

Among the Hymenoptera, 99.3% were ants (Formicidae). As a consequence of their colonial behavior, they fall in the traps in large numbers; therefore, the percentages of capture were calculated excluding Formicidae to better describe the dominance relationships between the captured groups. This revealed a shared sub-dominance between Araneae and Coleoptera, followed in magnitude by Orthoptera, Collembola, and Solifuga (Table 1, Figure 2). At the family level, the analysis showed a sub-dominance of six families (Sminthuridae, Tenebrionidae, Acrididae, Phloeothripidae, Carabidae, and Mummusidae) which represents more than 60% of the total catch. A complete description of the community at the order and family levels is given in Table 1.

**Figure 3.**
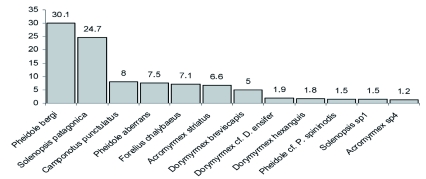
Relative abundance (larger than 1%) of ant species collected from Península Valdés (Patagonia, Argentina). High quality figures are available online.

**Figure 4.**
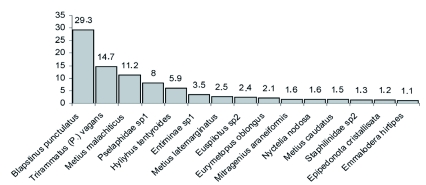
Relative abundance (larger than 1%) of beetle species collected from in Península Valdés (Patagonia, Argentina). High quality figures are available online.

Among the Formicidae caught, 75.1% belong to the Myrmicinae subfamily with *Pheidole bergi* Mayr and *Solenopsis patagonica* Emery being the most abundant species, representing more than 50% of the total captures (Figure 3). A complete description of the ant assemblage is given in Table 2. The most abundant families of beetles were Tenebrionidae and Carabidae, representing more than 75% of the total captures of this group, while the most numerous species were *Blapstinus punctulatus* Solier, *Trirammatus* (Plagioplatys) *vagans* (Dejean) and *Metius malachiticus* Dejean (Figure 4, Table 3).

**Figure 5.**
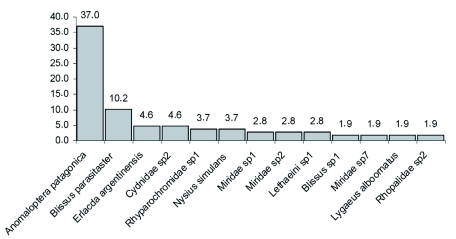
Relative abundance (larger than 1%) of true bug species collected from Península Valdés (Patagonia, Argentina). High quality figures are available online.

With respect to the true bug assemblage, the most numerous families were Oxicarenidae and Blissidae with more than 54% of the total captures of this group. The most abundant species was *Anomaloptera patagonica* Dellapé & Cheli (Figure 5); also found were *Valdesiana curiosa* Carpintero, Dellapé & Cheli (Miridae). Both taxa were very recently described as new based on specimens collected from this study. A complete description of the true bug community can be found in Table 4.

Abundance analysis: The distribution abundance model which best described the abundance data, both at the family and species levels, was the logarithmic series model (AIC fam: 202.231; AIC sp: 134.32). Also, this model best described the species abundances of ants (AIC: 138.551) and beetles (AIC: 134.318). The true bug species were equally well described both by the log series (AIC: 41.318) as well as the log normal series (AIC: 39.72) (Table 5).

In addition, excluding ants from the analysis increased the capacity of the logarithmic series model to describe the species abundance distribution of the community (AIC excluding ants: 513.668; AIC including ants: 652.527).

Diversity analysis: There was a significant increase of diversity (Shannon-Wiener index) at both the family and species levels when ants were excluded from the analysis (Hutchenson test: for the family level, t' = 101.494, p < 0.0001; for the species level, t' = 39.928, p < 0.0001) as well as an increase in the evenness of both taxonomical levels. At the species level, beetles were more diverse than ants (Hutchenson test; t' = 11.995, p < 0.0001). True bugs were equally as diverse as beetles (Hutchenson test, t' = 2.249, p = 0.026) and ants (Hutchenson test, t' = 1.645, p = 0.103). The Shannon species evenness measure was considerably high and similar among the three groups of species (Table 6).

**Table 2.**
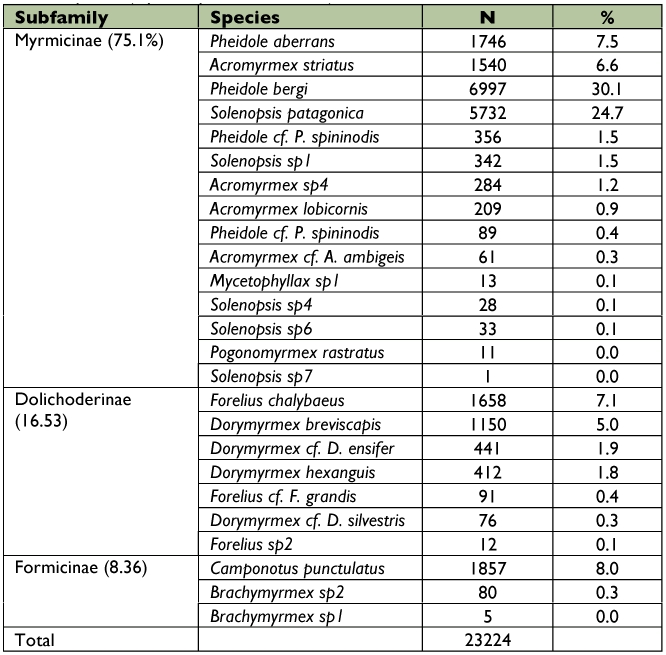
Abundance of ant species (Hymenoptera-Formicidae) in Península Valdés.

**Table 3.**
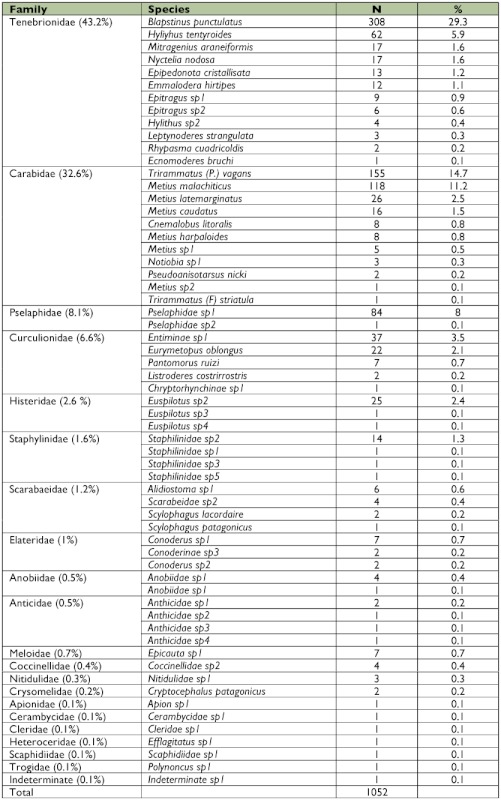
Abundance of beetle species (Coleoptera) in Península Valdés.

**Table 4.**
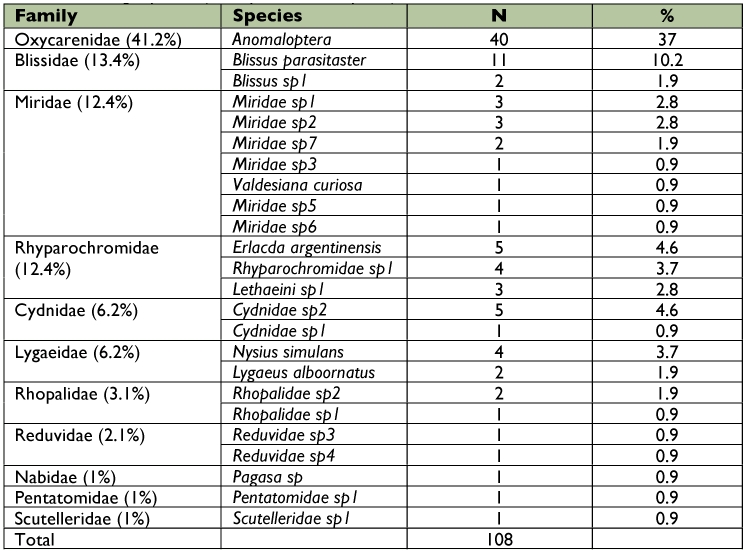
Abundance of true bugs species (Hemiptera-Heteroptera) in Península Valdés.

**Table 5.**
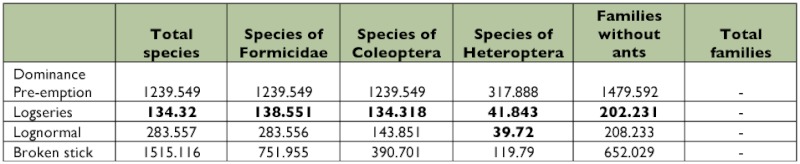
Fit to species abundances models (*p* values), Diversity (Shannon-Wiener index) and evenness values to family and species levels.

**Figure 6.**
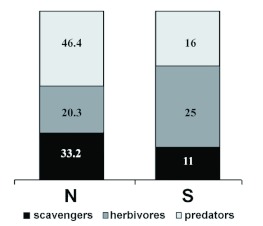
Relative abundance (%) and family richness of trophic guilds of ground-dwelling arthropods collected from Península Valdés (Patagonia.Argentina). High quality figures are available online.

Guild analysis: There was a significant difference among abundances of trophic guilds (*X^2^*_0.05; 2_ = 459.75; p < 0.001). The abundance of predators was greater than herbivores (*X^2^*_0.05; 1_ = 458.34; p < 0.001) and scavengers (*X^2^*_0.05; 1_ = 97.81; p < 0.001), while the abundances of scavengers were greater than herbivores (*X^2^*_0.05; 1_ = 139.64; p < 0.001). Family richness did not differ significantly among trophic guilds (*X^2^*_0.05; 2_ = 5.81; p = 0.0548) (Figure 6).

## Discussion

This is the first community study based on a planned and intensive sampling effort that describes the composition and structure of the ground-dwelling arthropod community of Península Valdés. The most important orders based on abundance were Hymenoptera, Coleoptera, and Araneae. The same community pattern was found in other arid areas of Argentina (Gardner et al. 1995; Molina et al. 1999; Lagos 2004), as well as in other regions of the world (Bromham et al. 1999; Seymour and Dean 1999). The three aforementioned orders are the most diverse and abundant in the world, and several authors considered them “hyper-diverse” taxa (Gibson et al. 1992; Martín-Piera and Lobo 2000; Lagos 2004).

The community was dominated by few abundant taxa at both family and species levels. Also, there were some groups with intermediate abundances and a large proportion of “rare” taxa for which very few individuals were caught. Therefore, the distribution of both species and family abundances were better described by the Logarithmic series model. This model depicts a system where some species could have arrived at an unsaturated habitat at randomly spaced intervals of time in order to occupy the remaining fractions of the niche hyperspace, thus having intermediate levels of niche preferences. Similarly, this model describes systems in which one or a few factors dominate the ecological relationships of the community and in which the intensity of migration between communities is important (Magurran 2004).

**Table 6.**
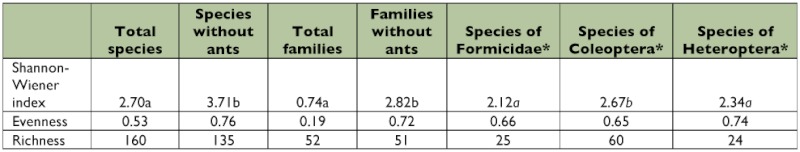
Diversity values of arthropod assemblages.

It is worth noting that, at the species level, taxa with remarkably different abundance, such as ants, beetles, and true bugs, were equally described by the logs series. Still, in the case of true bugs, which were adequately described both by the log and log normal series, this represents a special case of log normal distribution called “canonical.” Such pattern is a consequence of random niche separation every time a new species is incorporated into the assemblage (Magurran 2004). In this sense, these findings increase knowledge on niche segregation in general and on the invertebrate community structure of northeast Patagonia.

Ants are a central component of arthropod abundance in the study area, representing more than 80% of total captures. The contribution of *P. bergi* and *S. patagonica*, both well-known recruiting species, may explain such outstanding numbers. Still, excluding ants from analyses of the assemblages of northeast Patagonia lead to similar findings in terms of abundance patterns. Such consistency likely reflects the robustness of the model and its explanatory factors for the Patagonian arthropods.

In arid Patagonia, as in most deserts, the factors dominating the insect community structure are probably related to plants. Vegetation cover has shown to be correlated with diversity, dominance, and species abundance of ground-dwelling arthropods in other deserts (Crawford 1988; Seymour and Dean 1999). Vegetation structure usually provides the habitat template for the assembly of ground-dwelling arthropods in multitrophic communities by offering shelter, food resources, oviposition micro-sites, or refuge against predators (Dennis et al. 1998; Seymour and Dean 1999; Mazía et al. 2006). In turn, in northwest Patagonia, where there is a similar habitat to the one examined in this study, plant spatial structure has been shown to influence the activity of ground-dwelling ants and beetles (Farji-Brener et al. 2002; Folgarait and Sala 2002; Maz´a et al. 2006).

In addition, it should be considered that in Península Valdés sheep grazing has occurred since the late 19th century. Sheep grazing appears to have modified the vegetation and accelerated the soil degradation processes (Beeskow et al. 1995). These changes are generally referred to as changes in vegetation structure, diminishing their cover and exposing bare soil to erosive effects, which eventually leads to the fragmentation of the preexisting patches into smaller remnant patches (Bisigato and Bertiller 1997). Grazing, through its impact on vegetation, could be influencing observed arthropod communities.

From a trophic level approach, studies comparing protected areas versus grazed habitats in other arid areas from Argentina have found that arthropod communities were dominated by scavengers in protected sites and by predators in disturbed areas (Gardner 1995; Molina et al. 1999; Lagos 2004). In Península Valdés, the ground-dwelling arthropod community was dominated by predators, which suggests that sheep grazing could be one of the main variables modeling the arthropod assemblage structure. Predation could probably act as an important factor driving the distribution and abundances of surface-dwelling arthropods in this habitat (i.e., a top-down effect) and as such could be used as a key element in understanding the above-ground desert community structure.

This study found that the arthropod community of northern Patagonia had similar diversity values to those recorded in other arid areas of Argentina, such as the Chaco (Gardner et al. 1995; Molina et al. 1999) and the central Monte Desert (Lagos 2004). However, lower arthropod families and coleopteran species richness were found, as was smaller evenness at family and species levels. Reduced richness could be explained because of the lower temperatures present in Patagonia, which could constrain the number of species living there. In turn, a less even assemblage such as that found in this study suggests that the dominance of some species over others is greater than it is in other arid zones in northern Argentina. Species autoecological features coupled with a restrictive climate could explain why the community is dominated by a few species. For example, the most abundant beetle, *B. punctulatus* (Tenebrionidae), has a small body size that could allow them to hide into the soil fissures during extreme environmental periods. These features can also be observed in the true bug assemblage. For instance, *A. patagonica* is also small size and has wings like the elytra of coleoptera that enable it to tolerate extreme environmental conditions.

The adequate description by the same abundance distribution model both at the family and the species level suggests that the former can be a reasonable predictor of the subjacent abundance model in this community. This reduces costs in terms of time dedicated to taxonomic determination and is in accordance with previous work (e.g. Cagnolo et al. 2002). Using a higher taxonomic category than species level in community analysis has several advantages (see Gaston 2000), but it can be biased if the community has a fauna rich in endemisms (Samways et al. 1996).

The results obtained in this study could be extended to all of arid Patagonia, due to similar environmental conditions in the area. This work not only improves the knowledge of the composition, taxonomy, and trophic structure of ground-dwelling arthropod communities in arid Patagonian habitats, but also increases the taxonomic knowledge of Hemiptera through the discoveries of new genera and two new species very recently described as new based on material recovered from this survey (see Dellapé and Cheli 2007; Carpintero et al. 2008). Additionally, it is necessary to place the results of this study within a conservation context because the richness and composition of a community of ground-dwelling arthropods can be taken as a reflection of the biotic and structural diversity of whole terrestrial ecosystems (Iannacone and Alvariño 2006). Because of its abundance, diverse behaviors, and ecological interactions, the development of new lines of research to elucidate the variables controlling the main ecological aspects of grounddwelling arthropods will contribute significantly to the knowledge and functioning of arid Patagonian ecosystems. It also may help to create and assess management and conservation tools for the arid terrestrial ecosystem.
